# German translation and initial measurement properties of the Q-TFA in transfemoral bone-anchored prosthesis users

**DOI:** 10.1186/s12891-026-10490-z

**Published:** 2026-09-19

**Authors:** Harun Hawi, Alexander Ranker, Alaa Sharfo, Emmanouil Liodakis, Marcel Orth, Sebastian Schreiber, Marcus Örgel

**Affiliations:** 1 Department of Trauma, Hand and Reconstructive Surgery, Saarland University, Homburg, Germany; 2 Department of Rehabilitation und Sports Medicine, Hannover Medical School, Hannover, Germany; 3 Institute for General Practice and Palliative Care, Hannover Medical School, Hannover, Germany

**Keywords:** Transfemoral amputation, Bone-anchored prosthesis, Patient-reported outcome measure, Q-TFA, Cross-cultural adaptation, Measurement properties

## Abstract

**Background:**

To translate and cross-culturally adapt the Questionnaire for Persons with a Transfemoral Amputation (Q-TFA) into German and evaluate initial measurement properties in adults with unilateral transfemoral amputation using a transcutaneous bone-anchored prosthesis.

**Methods:**

Sixty-one participants completed the German Q-TFA and comparator patient-reported outcome measures at baseline and after 12–15 days. The original four-score Q-TFA structure was retained: Prosthetic Use, Prosthetic Mobility, Problem, and Global scores. Test-retest reliability, measurement error, internal consistency of the Problem Score, floor and ceiling effects, and hypothesis-based construct validity were assessed.

**Results:**

The 61 participants (42 male, 19 female; mean age 56.2 ± 11.7 years) used bone-anchored transfemoral prostheses; trauma was the etiology in 74%. Baseline mean ± SD scores were 82.1 ± 29.2 for Prosthetic Use, 52.0 ± 10.0 for Prosthetic Mobility, 21.3 ± 16.6 for Problems, and 65.4 ± 18.6 for Global. ICCs ranged from 0.960 to 1.000. The Problem Score had high internal consistency (Cronbach’s α = 0.955), no score showed a substantial floor or ceiling effect, and all six nonredundant construct-validity hypotheses were confirmed.

**Conclusions:**

The German Q-TFA, scored according to its original four-score structure, showed high short-interval test-retest reliability and supportive construct-validity findings in this selected bone-anchored prosthesis cohort. Structural validity, patient-based content validity, responsiveness, and interpretability remain to be established before the instrument is used to infer clinically important change or for formal cross-center benchmarking.

**Supplementary Information:**

The online version contains supplementary material available at https://doi.org/10.1186/s12891-026-10490-z.

## Background

Transfemoral amputation (TFA) can substantially affect mobility, participation, body image, pain, psychological well-being, and health-related quality of life. Quality of life after lower-limb amputation is multifactorial: perceived prosthetic mobility, prosthesis-related problems, depression, social support and participation, comorbidity, and age have all been associated with patient-reported quality of life [1]. In bone-anchored prosthesis users, relief from socket-related limitations may coexist with stoma or implant complications, infection, mechanical events, and rehabilitation burden [2, 3]. Generic health measures such as the EQ-5D-5 L remain important, but no single measure captures this complete range of outcomes [4–5].

Available prosthesis-related PROMs target different constructs. The German PMQ 2.0 is a 12-item measure of prosthetic mobility, and the German SAT-PRO is a 15-item measure of prosthesis satisfaction; a single total score from each was used in this study [6–7]. These are not the only relevant amputee-specific instruments: for example, the internationally used Prosthesis Evaluation Questionnaire (PEQ) provides a broader multiscale assessment of prosthesis-related quality of life [8]. The Q-TFA was selected not because alternatives were unavailable, but because it was developed specifically for persons with TFA, combines use, mobility, amputation- and prosthesis-related problems, and global situation, and is established in bone-anchored prosthesis research [9–15]. It produces four established 0-100 scores: Prosthetic Use, Prosthetic Mobility, Problem, and Global. Higher Use, Mobility, and Global scores indicate better outcomes, whereas a higher Problem Score indicates more severe problems.

The initial measurement-property study was conducted in 156 users of socket-suspended transfemoral prostheses, although the Q-TFA was developed to evaluate both socket and bone-anchored prosthesis users and changes in prosthetic attachment [9]. Subsequent Swedish, Dutch, French, and North American bone-anchored cohorts have used one or more of the original Q-TFA scores [2–15].

Reporting conventions have not been fully uniform. Swedish OPRA studies generally report all four scores, with some later reports reversing only the direction of the Problem Score so that higher values consistently represent better outcomes [2, 3]. Dutch studies have often selected the Use and Global scores for their specific study aims rather than modifying the dimensional structure [10]. These directional and selective-reporting choices should be distinguished from creating a new score.

A German Q-TFA has previously been used in comparative and related bone-anchored prosthesis research [14–15, 16], but its cross-cultural adaptation process and measurement properties have not been reported in detail. This gap is relevant because heterogeneity in outcome selection limits evidence synthesis [17–18], while international initiatives seek more consistent outcome reporting in bone-anchored prosthesis care [19–20]. The recent Brazilian Portuguese adaptation further illustrates the need for transparent language-specific methods [21].

The aim of this study was therefore to document the German translation and cross-cultural adaptation of the Q-TFA and to evaluate initial measurement properties in adults with unilateral TFA using a transcutaneous bone-anchored prosthesis. The evaluation was restricted to short-interval test-retest reliability, measurement error, floor and ceiling effects, internal consistency of the Problem Score, and hypothesis-based construct validity. The sample size and two-occasion design, without an external transition anchor, were not suitable for adequately powered structural-validity, responsiveness, or minimal-important-change analyses; these properties were therefore outside the study scope and are addressed as limitations [22–23].

## Methods

### Study design and setting

This cross-sectional measurement-property study included a 12-15-day retest component. Participants were treated and followed at Hannover Medical School, Germany. The study was approved by the Ethics Committee of Hannover Medical School (8937_BO_K_2020). This was a non-commercial academic study.

### Participants

Adults with unilateral TFA actively using a transcutaneous bone-anchored prosthesis were eligible when at least 6 months had elapsed since their last osseointegration surgery. This pragmatic lower threshold was chosen to exclude the immediate postoperative period and early prosthetic rehabilitation; it was not intended to define a universally stable postoperative endpoint. The date of definitive prosthesis provision and completion of rehabilitation were not recorded separately. Exclusion criteria were bilateral amputation, an acute complication requiring revision surgery, insufficient German-language proficiency, and non-consent. The available complete-pair sample determined the study size; no a priori sample-size calculation was performed, and precision is reported using 95% confidence intervals.

### Questionnaire provenance

All questionnaires used were based on previously published instruments; none was newly developed for this study. The original Q-TFA and scoring system were published by Hagberg et al. [9]. The present study documents its German translation and cross-cultural adaptation and evaluates initial measurement properties, rather than developing a new questionnaire.

The 12-item German PMQ 2.0 was previously translated, cross-culturally adapted, and psychometrically evaluated by Ranker et al., with additional Rasch validation reported separately [6, 24]. The 15-item German SAT-PRO was previously translated, cross-culturally adapted, and psychometrically evaluated by Wahl et al. [7]. The EQ-5D-5 L was developed and initially tested by Herdman et al. [4], and Hinz et al. reported psychometric properties and normative values for the German general population [25]. The EQ VAS is the self-rated health component of the EQ-5D-5 L, not a separate study-developed questionnaire [4, 25]. Use of the EQ-5D-5 L, including the EQ VAS, was authorized by the EuroQol Research Foundation (registration no. 87745).

### Translation

The Q-TFA was not developed for this study; the original instrument and scoring system were published by Hagberg et al. [9]. The German version was produced using established forward/back-translation methods for self-report measures [26]. Two native German-speaking physicians independently performed forward translations. An expert committee comprising two orthopaedic surgeons, one rehabilitation specialist, and one professional translator reconciled the translations. Two independent back-translations were compared with the original instrument, and discrepancies were resolved by consensus. Formal patient cognitive debriefing was not performed. The evaluation was planned and reported with reference to COSMIN methodology [22–23].

### Outcome measures and scoring

The published Q-TFA scoring structure was retained [9, 27]. Four scores were transformed to 0-100: Prosthetic Use (2 items; higher=better), Prosthetic Mobility (19 items; higher=better), Problem (30 paired items; higher=worse), and Global (3 items; higher=better). The Mobility Score is the mean of the walking-aids, capability, and walking-habits subscores. Each Problem item pairs the extent of a problem with its impact on quality of life; the paired response is combined within the single validated Problem Score. At least 15 paired Problem items are required. No separate severity or quality-of-life-impact dimension was created. The corrected German questionnaire supplied with the revision displays the full G1 and G2 response ranges and the original four-week recall period for G8 and G9 (Supplementary File 2).

Comparator instruments were the 12-item German PMQ 2.0 and 15-item German SAT-PRO as construct-specific prosthesis measures [6–7], and the EQ-5D-5 L profile with EQ VAS as generic health comparators [4–5]. A single PMQ 2.0 total score and a single SAT-PRO total score were transformed to 0-100, with higher scores indicating better mobility or satisfaction. In addition to EQ VAS, an exploratory non-preference-based EQ-5D-5 L profile severity summary was calculated by summing the five dimension levels and rescaling to 0-100 (higher=worse). Comparator characteristics and baseline scores are presented in Table 2.

### Statistical analysis

Descriptive statistics are reported as mean ± SD. Q-TFA scores are reported to one decimal place, ICCs and 95% confidence intervals to three decimals, and ANOVA mean squares, SEM, and MDC95 to two decimals. Floor and ceiling effects were the proportions at the theoretical minimum and maximum; values > 15% were considered substantial [28]. All reliability and measurement-error analyses used 61 complete retest pairs. One participant had one unanswered paired Problem item at baseline but exceeded the original minimum of 15 pairs and was retained for Problem scoring; no value was imputed.

Internal consistency of the 30 paired Problem items was assessed using Cronbach’s α in the 60 participants with complete item-level data [28]. Test-retest reliability was assessed with a two-way random-effects, absolute-agreement ICC for single measurements (ICC(2,1)) [29]. ANOVA mean squares for participants (MSR), occasions (MSC), and residual error (MSE) are reported. Measurement error was quantified as SEM=√MSE and MDC95 = 1.96×√2×SEM [30]. The MDC95 represents statistically detectable change and was not interpreted as clinically important change.

Construct validity was assessed using Spearman correlations between the original Q-TFA scores and comparator measures. The eight component-level relationships listed in the submitted analysis contained duplicate hypotheses for the two separately reported components of the original Problem Score. After restoration of the validated four-score structure, these were consolidated into six nonredundant score-level hypotheses without changing the expected directions or magnitude thresholds: Prosthetic Use was expected to correlate positively with PMQ 2.0 (ρ ≥ 0.50) and EQ VAS (ρ ≥ 0.30); the Problem Score was expected to correlate positively with EQ-5D-5 L profile severity (ρ ≥ 0.50) and negatively with SAT-PRO (ρ≤-0.50); and the Global Score was expected to correlate positively with EQ VAS and SAT-PRO (both ρ ≥ 0.30). The rationale, observed correlations, and confirmation status are detailed in Supplementary Table S1. Confirmation of at least 75% was considered supportive [22–23]. Correlations were interpreted as strong when |ρ|≥0.50 and moderate when 0.30≤|ρ|<0.50. Exploratory sex-stratified baseline comparisons used the Mann-Whitney U test.

## Results

### Participant characteristics

**Fig. 1:**
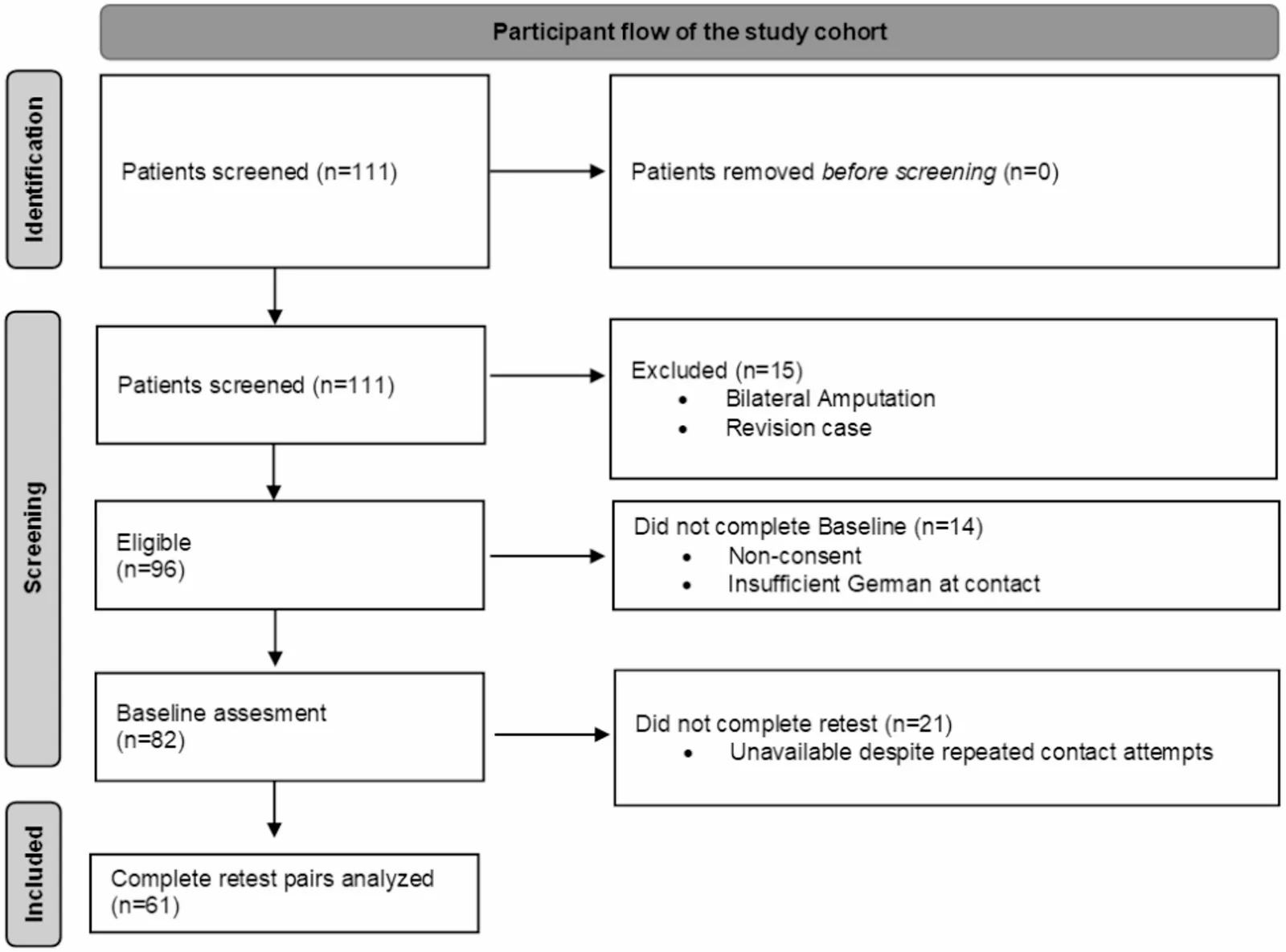
Participant flow (screening, eligibility, baseline assessment, and 12–15‑day retest completion)

Of 111 screened patients, 96 met clinical eligibility criteria, 82 completed baseline assessment, and 61 completed the 12-15-day retest and formed the analysis sample. Patients not meeting eligibility criteria were excluded primarily because of bilateral amputation, insufficient German-language proficiency, or an acute complication requiring revision surgery. Among eligible patients who did not complete baseline assessment, reasons included non-consent or insufficient German-language proficiency identified at contact. Of the 82 baseline participants, 21 did not complete retest because they were unavailable despite repeated contact attempts. The 61 analyzed participants comprised 42 men and 19 women; mean age was 56.2 ± 11.7 years and mean BMI was 28.5 ± 6.2 kg/m². All actively used a transcutaneous bone-anchored transfemoral prosthesis and were at least 6 months from their last osseointegration surgery. A formal community-walker or functional ambulation classification was not collected, so the proportion of community walkers cannot be reported. The observed Prosthetic Use and Mobility scores describe a range of self-reported prosthetic use and mobility within the cohort. Trauma was the etiology in 45/61 participants (74%); other etiologies are shown in Table 1. Participant flow is shown in Fig. 1.

**Table 1:** Etiology of transfemoral amputation

Etiology	*n*	%
Trauma	45	74
Iatrogenic	5	8
Infection	5	8
Tumor	4	7
Peripheral artery disease	2	3

Baseline comparator scores are summarized in Table 2.

**Table 2:** Comparator patient-reported outcome measures and baseline scores

Instrument	Construct	Possible range	Direction	Mean (SD)
EQ VAS	Self-rated health	0-100	Higher=better	80.8 (15.9)
EQ-5D-5 L profile severity*	Generic health problems	0-100	Higher=worse	19.7 (19.1)
PMQ 2.0	Prosthetic mobility	0-100	Higher=better	57.6 (24.0)
SAT-PRO	Prosthesis satisfaction	0-100	Higher=better	83.7 (24.0)

*Exploratory non-preference-based profile summary

### Q-TFA scores, floor/ceiling effects, and internal consistency

Baseline and retest Q-TFA scores are summarized in Table 3. No original Q-TFA score exceeded 15% at either endpoint. Internal consistency of the 30 paired Problem items was high (Cronbach’s α = 0.955; *n* = 60 complete item sets).

**Table 3:** Original Q-TFA scores at baseline and retest, change scores, and baseline floor/ceiling percentages

Score (0-100)	Direction	Baseline	Retest	Δ (retest-baseline)	Floor/Ceiling (baseline)
Prosthetic Use	Higher=better	82.1 (29.2)	82.2 (29.3)	0.0 (0.2)	3.3% / 0.0%
Prosthetic Mobility	Higher=better	52.0 (10.0)	51.7 (9.6)	-0.3 (2.8)	0.0% / 0.0%
Problem	Higher=worse	21.3 (16.6)	21.8 (16.2)	0.4 (0.9)	0.0% / 0.0%
Global	Higher=better	65.4 (18.6)	65.3 (18.2)	-0.1 (1.9)	0.0% / 0.0%

### Test–retest reliability and measurement error

Agreement and measurement-error estimates for the four original Q-TFA scores are presented in Table 4. Short-interval reliability was high for all scores (ICC range 0.960-1.000). The very small error estimates for Prosthetic Use should be interpreted in the context of a short retest interval and a clinically stable, selected cohort.

**Table 4:** Test-retest reliability and measurement error for the four original Q-TFA scores

Score	*n*	ICC	CI low	CI high	MSR	MSC	MSE	SEM	MDC95
Prosthetic Use	61	1.000	1.000	1.000	1711.08	0.06	0.029	0.17	0.47
Prosthetic Mobility	61	0.960	0.934	0.976	189.25	3.10	3.871	1.97	5.45
Problem	61	0.998	0.997	0.999	537.18	5.83	0.427	0.65	1.81
Global	61	0.995	0.992	0.997	674.24	0.57	1.727	1.31	3.64

*n* = 61 complete pairs

*ICC* intraclass correlation coefficient, *CI* confidence interval, *MSR* mean square for rows/participants, *MSC* mean square for columns/occasions, *MSE* mean square error, *SEM* standard error of measurement, *MDC95* minimal detectable change at the 95% confidence level

All six nonredundant score-level hypotheses were confirmed (Supplementary Table S1). Prosthetic Use correlated strongly with PMQ 2.0 (ρ = 0.69, 95% CI 0.53 to 0.80) and moderately with EQ VAS (ρ = 0.47, 95% CI 0.24 to 0.64). The Problem Score correlated strongly with EQ-5D-5 L profile severity (ρ = 0.66, 95% CI 0.49 to 0.78) and inversely with SAT-PRO (ρ=-0.73, 95% CI -0.83 to -0.58). The Global Score correlated strongly with EQ VAS (ρ = 0.51, 95% CI 0.29 to 0.67) and moderately with SAT-PRO (ρ = 0.40, 95% CI 0.17 to 0.60).

In exploratory baseline analyses, women had a lower mean Global Score than men (60.1 ± 19.9 versus 67.9 ± 17.6; *p* = 0.047). Prosthetic Use, Prosthetic Mobility, and Problem scores did not differ statistically by sex.

## Discussion

This study documents the German cross-cultural adaptation of the Q-TFA and provides initial measurement-property evidence in 61 adults with unilateral TFA using bone-anchored prostheses. When the original four-score structure was applied, the German Q-TFA showed high short-interval reliability, small measurement error, no substantial floor or ceiling effects, high internal consistency of the Problem Score, and construct-validity correlations in the expected directions.

An important correction in this revision is the restoration of the original scoring structure. The Q-TFA was neither developed nor structurally modified in this study. The submitted manuscript had presented the two components of each paired Problem item - problem extent and quality-of-life impact - as separate analytical scores and had reversed and renamed the Prosthetic Use Score. This presentation could reasonably be interpreted as a new dimensional model. Because a true split would require an adequately powered structural-validity study, we did not attempt to justify it post hoc. Instead, all primary analyses were repeated using the four validated scores described by Hagberg et al. [9].

This approach is consistent with international practice. Swedish OPRA cohorts report Prosthetic Use, Prosthetic Mobility, Problem, and Global scores [2, 3, 27]. The ten-year report reversed the direction of the Problem Score for presentation so that improvement had a common positive direction, but did not alter its dimensional content [3]. Dutch investigations have often selected Prosthetic Use and Global scores according to their study questions [10], while German comparative work reported the original domains [16]. Selective reporting and directional reversal can complicate comparison if not explicit, but neither constitutes evidence for splitting the Problem Score. We therefore retained the original names and directions and state them in every table.

The population difference from the original measurement-property study also warrants caution. Hagberg et al. evaluated 156 socket-prosthesis users recruited from multiple community and clinical sources; 92% had nonvascular etiologies [9]. The present cohort consisted only of established, unilateral bone-anchored prosthesis users treated at one specialist center and was trauma-dominant. Surgical selection, successful completion of rehabilitation, and exclusion of bilateral users or acute revision cases may produce a more homogeneous clinical spectrum. ICCs are sample-dependent: restricted between-person variability may reduce ICCs, whereas clinical stability, a short retest interval, and highly reproducible response patterns may increase apparent agreement. The very high estimates observed here should not be generalized uncritically to socket users, early rehabilitation, or broader dysvascular populations.

The restored Prosthetic Use mean of 82.1 is directionally consistent with high wear time in established bone-anchored users. The Global Score of 65.4 lies between the Dutch pre-implant median of 33 and five-year median of 75 [10]. Such comparisons remain contextual because the present cohort was cross-sectional and postoperative, whereas many international studies compare a preoperative socket baseline with longitudinal postoperative follow-up [2–11]. Different rehabilitation stages, eligibility criteria, and summary statistics further limit direct numerical benchmarking.

No original score showed a substantial floor or ceiling effect, supporting score dispersion within this sample. Cronbach’s α for the Problem Score was 0.955. Although this is consistent with strong interrelation among paired problem items and with the original study [9], values near 0.95 can also indicate item redundancy. The Q-TFA was developed with both clinimetric and psychometric considerations, and a high α alone does not establish unidimensionality. Structural validity and item-level performance therefore remain important future questions [22–23, 24].

Test-retest reliability was high, with ICCs from 0.960 to 1.000. The original socket-user study reported ICCs of approximately 0.89–0.94 and larger measurement error [9]. Differences may reflect the sample, scoring, administration, and stability criteria rather than superior intrinsic performance of the German version. In particular, the present 12-15-day interval was short, clinical stability was inferred from the study setting rather than verified with a dedicated transition item, and recall cannot be excluded.

The SEM and MDC95 quantify random measurement error under the present conditions. They do not demonstrate responsiveness and do not identify clinically important change. For example, an observed difference exceeding the Global Score MDC95 of 3.6 points would be unlikely to arise from measurement error alone in this sample, but it cannot be interpreted as important to patients without an anchor-based threshold. A 17-point anchor-based estimate has been reported in a contemporary bone-anchored cohort [13], but transportability to this German cohort has not been established.

Construct-validity findings were supportive and aligned with the six prespecified relationships. The strong association between Prosthetic Use and PMQ 2.0 and the moderate association with EQ VAS are compatible with prosthetic use reflecting both mobility and broader perceived health. The Problem Score showed the expected positive association with generic health-profile severity and negative association with prosthesis satisfaction. The Global Score showed the expected positive associations with self-rated health and satisfaction. These patterns support convergent construct validity and agree with concurrent-validity evidence in bone-anchored users [12], but shared content between PROMs and confirmation in a single cross-sectional cohort do not constitute complete validation.

The Q-TFA should also not be interpreted as a comprehensive quality-of-life instrument. Its four scores cover prosthetic use, mobility, a set of paired amputation- and prosthesis-related problems, and global situation; individual Problem items include selected pain and appearance-related concerns. It does not comprehensively assess multidimensional pain, body image, depression, social support, comorbidity, or participation, all of which may influence quality of life after lower-limb amputation [1]. Bone-anchored treatment adds benefits and burdens not fully summarized by a single PROM, including relief from socket problems as well as stoma, infection, implant, and mechanical complications [2, 3]. Generic health measures and targeted instruments should therefore complement, rather than be replaced by, the Q-TFA. A German-language Q-TFA has been used previously in bone-anchored prosthesis research [14–15, 16]; the present contribution is transparent documentation of its translation and initial measurement properties, not creation of a new questionnaire.

Limitations include the single-center design, small sample, trauma-dominant and surgically selected cohort, absence of formal patient cognitive debriefing, and lack of an external stability criterion at retest. The 6-month postsurgical eligibility threshold excluded early recovery but was pragmatic rather than a validated stability threshold; the dates of definitive prosthesis provision and rehabilitation completion were not recorded separately. Formal community-walker or functional ambulation status was not collected. One baseline Problem pair was missing for one participant; the score remained calculable under the original minimum-item rule, while α used 60 complete cases. The exploratory sex comparison was underpowered and susceptible to type I error. Structural validity, measurement invariance, responsiveness, minimal important change, and agreement across modes of administration were not evaluated. These limitations restrict generalizability and preclude claims about individual change monitoring or formal cross-center benchmarking.

Future work should include patient cognitive interviews, adequately powered structural analyses, prespecified responsiveness hypotheses with external anchors, and multicenter testing across socket and bone-anchored users with more diverse etiologies. Until then, the present results support only the initial reliability and construct-validity evidence demonstrated under the study conditions.

## Conclusions

In 61 adults with established bone-anchored transfemoral prostheses, the German Q-TFA scored according to the original four-score structure showed high 12-15-day test-retest reliability, small measurement error, no substantial floor or ceiling effects, and supportive hypothesis-based construct validity. Further work on patient-based content validity, structural validity, responsiveness, and interpretability is required before clinically important change or formal cross-center comparability can be inferred.

## Supplementary Information

Supplementary Material 1.

## Data Availability

The datasets used and/or analysed during the current study are available from the corresponding author on reasonable request.

## Abbreviations

ANOVA: Analysis of variance
BALLCOS: Bone-Anchored Lower Limbs Core-Outcome Set
BMI: Body mass index
CI: Confidence interval
COMET: Core Outcome Measures in Effectiveness Trials
COSMIN: Consensus-based Standards for the selection of health Measurement Instruments
EQ-5D-5L: EuroQol five-dimension five-level questionnaire
EQ VAS: EuroQol visual analogue scale
ICC: Intraclass correlation coefficient
ISPO: International Society for Prosthetics and Orthotics
MCID: Minimal clinically important difference
MDC95: Minimal detectable change at the 95% confidence level
MSC: Mean square for columns/time
MSE: Mean square error
MSR: Mean square for rows/subjects
PMQ 2.0: Prosthetic Mobility Questionnaire 2.0
PROM: Patient-reported outcome measure
PROMIS: Patient-Reported Outcomes Measurement Information System
Q-TFA: Questionnaire for Persons with a Transfemoral Amputation
QoL: Quality of life
SAT-PRO: Satisfaction with Prosthesis Questionnaire
SEM: Standard error of measurement
SIGBAL: Special Interest Group for Bone Anchored Limbs
TFA: Transfemoral amputation
TOPS: Transcutaneous osseointegrated prosthetic systems

## References

[1] Asano M, Rushton P, Miller WC, Deathe BA. Predictors of quality of life among individuals who have a lower limb amputation. Prosthet Orthot Int. 2008;32(2):231–43. 10.1080/03093640802024955. 18569891

[2] Brånemark R, Berlin Ö, Hagberg K, Bergh P, Gunterberg B, Rydevik B. A novel osseointegrated percutaneous prosthetic system for the treatment of patients with transfemoral amputation: a prospective study of 51 patients. Bone Joint J. 2014;96–B(1):106–13. 10.1302/0301-620X.96B1.31905. 24395320

[3] Hagberg K, Ghasemi Jahani SA, Omar O, Thomsen P. Osseointegrated prostheses for the rehabilitation of patients with transfemoral amputations: a prospective ten-year cohort study of patient-reported outcomes and complications. J Orthop Translat. 2023;38:56–64. 10.1016/j.jot.2022.09.004. PMC9588992 36313974

[4] Herdman M, Gudex C, Lloyd A, Janssen M, Kind P, Parkin D, Bonsel G, Badia X. Development and preliminary testing of the new five-level version of EQ-5D (EQ-5D-5L). Qual Life Res. 2011;20(10):1727–36. 10.1007/s11136-011-9903-x. PMC3220807 21479777

[5] Ludwig K, von der Graf JM, Greiner W. German value set for the EQ-5D-5L. PharmacoEconomics. 2018;36(6):663–74. 10.1007/s40273-018-0615-8. PMC5954069 29460066

[6] Ranker A, Örgel M, Schiller J, Egen C, Ranker AH, Greitemann B, Gutenbrunner C. Translation, adaptation, reliability and validity of the German version of the Prosthesis Mobility Questionnaire 2.0 (PMQ) in patients with major lower limb amputation. Rehabilitation (Stuttg). 2021;60(6):374–83. 10.1055/a-1506-7603. 34293808

[7] Wahl B, Gutenbrunner C, Greitemann B, Örgel M, Somoza López D, Schiller J, Ranker A. The German version of the Satisfaction with Prosthesis Questionnaire: translation, adaptation, reliability, and validity in adults with major lower-limb amputation. J Prosthet Orthot. 2023;35(1):44–54. 10.1097/JPO.0000000000000388.

[8] Legro MW, Reiber GD, Smith DG, del Aguila M, Larsen J, Boone D. Prosthesis Evaluation Questionnaire for persons with lower limb amputations: assessing prosthesis-related quality of life. Arch Phys Med Rehabil. 1998;79(8):931–8. 10.1016/S0003-9993(98)90090-9. 9710165

[9] Hagberg K, Brånemark R, Hägg O. Questionnaire for Persons with a Transfemoral Amputation (Q-TFA): initial validity and reliability of a new outcome measure. J Rehabil Res Dev. 2004;41(5):695–706. 10.1682/JRRD.2003.11.0167. 15558399

[10] Reetz D, Atallah R, Mohamed J, van de Meent H, Frölke JPM, Leijendekkers R. Safety and Performance of Bone-Anchored Prostheses in Persons with a Transfemoral Amputation: A 5-Year Follow-up Study. J Bone Joint Surg Am. 2020;102(15):1329–35. 10.2106/JBJS.19.01169. 32769599

[11] Thouvenin C, Bertrand–Marchand M, Klotz R, Puges M, Fabre T, Delgove A. Bone–anchored prostheses for lower limb amputation in a French cohort with 1–15 years of follow–up: implant survival rates, mechanical complications, and reported outcomes. Eur J Orthop Surg Traumatol. 2024;34(2):885–92. 10.1007/s00590-023-03738-0. 37750975

[12] Sabharwal S, Skolasky RL, Souza JM, Potter BK, Forsberg JA. Concurrent Validity of Q-TFA With PROMIS and Prosthetic Wear Time in Transfemoral Amputees. J Orthop Trauma. 2022;36(7):361–5. 10.1097/BOT.0000000000002325. 35727004

[13] Potter BK, Rivera JA, Anderson AB, Souza JM, Forsberg JA. What Functional Outcomes Can Be Expected With Osseointegrated Prostheses in Transfemoral Amputations? Clin Orthop Relat Res. 2025;483(3):513–23. 10.1097/CORR.0000000000003267. PMC11827995 39898879

[14] Örgel M, Elareibi M, Graulich T, Krettek C, Neunaber C, Aschoff HH, Ranker A, Winkelmann M. Osseoperception in transcutaneous osseointegrated prosthetic systems (TOPS) after transfemoral amputation: a prospective study. Arch Orthop Trauma Surg. 2023;143(2):603–10. 10.1007/s00402-021-04099-1. 34345935

[15] Örgel M, Petri M, Ranker A, Wirries N, Graulich T, Krettek C, Winkelmann M, Aschoff HH. Management, outcome, and novel classification system of periprosthetic fractures in patients with transcutaneous osseointegrated prosthetic systems (TOPS)-a retrospective cohort analysis. Arch Orthop Trauma Surg. 2021. 10.1007/s00402-021-03826-y. PMC9217873 33675390

[16] Pospiech PT, Wendlandt R, Aschoff HH, Ziegert S, Schulz AP. Quality of life of persons with transfemoral amputation: comparison of socket prostheses and osseointegrated prostheses. Prosthet Orthot Int. 2021;45(1):20–5. 10.1177/0309364620948649. 33834741

[17] Al Muderis MM, Lu WY, Li JJ, Kaufman K, Orendurff M, Highsmith MJ, Lunseth PA, Kahle JT. Clinically relevant outcome measures following limb osseointegration: systematic review of the literature. J Orthop Trauma. 2018;32(2):e64–75. 10.1097/BOT.0000000000001031. 29373379

[18] Portnoff BS, Byrne RA, Hao KA, Gutowski CT, Lin Y, Hoffman RA, Fedorka CJ, King JJ, Green A, Paxton ES. Trends in reported outcomes and patient reported outcome measures (PROMs) in humeral shaft fracture literature: a systematic review. Eur J Orthop Surg Traumatol. 2024;34(6):2859–70. 10.1007/s00590-024-04039-w. 39009710

[19] International Society for Prosthetics and Orthotics (ISPO). Special Interest Group for Bone Anchored Limbs (SIGBAL). Available at: https://www.ispoint.org/special-interest-groups/bone-anchored-limbs/. Accessed 15 Apr 2026.

[20] COMET Initiative. Delphi study for Bone-Anchored Lower Limbs Core-Outcome Set. COMET database (Study ID 3435). Current stage: ongoing; April 2025–July 2025. Accessed 24 Feb 2026.

[21] Batista PKA, Silva SL, da da, Silva RAC, Ferreira TLB. Tradução e adaptação transcultural do Questionnaire for Persons with a Transfemoral Amputation (Q-TFA), para a língua portuguesa do Brasil. Acta Fisiatr. 2024;31(4):241–9. 10.11606/issn.2317-0190.v31i4a220038.

[22] Prinsen CAC, Mokkink LB, Bouter LM, Alonso J, Patrick DL, de Vet HCW, Terwee CB. COSMIN guideline for systematic reviews of patient-reported outcome measures. Qual Life Res. 2018;27(5):1147–57. 10.1007/s11136-018-1798-3. PMC5891568 29435801

[23] Terwee CB, Prinsen CAC, Chiarotto A, Westerman MJ, Patrick DL, Alonso J, Bouter LM, de Vet HCW, Mokkink LB. COSMIN methodology for evaluating the content validity of patient-reported outcome measures: a Delphi study. Qual Life Res. 2018;27(5):1159–70. 10.1007/s11136-018-1829-0. PMC5891557 29550964

[24] Ranker A, Gutenbrunner C, Eckhardt I, Giordano A, Burger H, Franchignoni F. Rasch validation and comparison of the German versions of the Locomotor Capabilities Index-5 and Prosthetic Mobility Questionnaire 2.0 in lower-limb prosthesis users. Int J Rehabil Res. 2021;44(3):233–40. 10.1097/MRR.0000000000000478. 34034290

[25] Hinz A, Kohlmann T, Stöbel-Richter Y, Zenger M, Brähler E. The quality of life questionnaire EQ-5D-5L: psychometric properties and normative values for the general German population. Qual Life Res. 2014;23(2):443–7. 10.1007/s11136-013-0498-2. 23921597

[26] Beaton DE, Bombardier C, Guillemin F, Ferraz MB. Guidelines for the process of cross-cultural adaptation of self-report measures. Spine (Phila Pa 1976). 2000;25(24):3186–91. 10.1097/00007632-200012150-00014. 11124735

[27] Hagberg K, Brånemark R. One hundred patients treated with osseointegrated transfemoral amputation prostheses: rehabilitation perspective. J Rehabil Res Dev. 2009;46(3):331–44. 19675986

[28] Terwee CB, Bot SDM, de Boer MR, et al. Quality criteria were proposed for measurement properties of health status questionnaires. J Clin Epidemiol. 2007;60(1):34–42. 10.1016/j.jclinepi.2006.03.012. 17161752

[29] Shrout PE, Fleiss JL. Intraclass correlations: uses in assessing rater reliability. Psychol Bull. 1979;86(2):420–8. 10.1037/0033-2909.86.2.420. 18839484

[30] de Vet HCW, Terwee CB, Mokkink LB, Knol DL. Measurement in Medicine: A Practical Guide. Cambridge University Press; 2011.

