# Supplementary material for: German translation and initial measurement properties of the Q-TFA in transfemoral bone-anchored prosthesis users
Source: BMC Musculoskelet Disord. 2026 Sep 19;27:829. doi: 10.1186/s12891-026-10490-z (PMC13640152; doi:10.1186/s12891-026-10490-z)
Supplement: Supplementary file 1 [file 12891_2026_10490_MOESM1_ESM.docx]

**SUPPLEMENTARY METHODS AND TABLE S1**

# Supplementary scoring methods

The Analysis uses the original four-score structure described by Hagberg et al.: Prosthetic Use, Prosthetic Mobility, Problem, and Global scores. Scores range from 0 to 100. Higher values indicate better outcomes for Use, Mobility, and Global; higher values indicate worse outcomes for Problem. The two questions paired for each of the 30 Problem items (problem extent and quality-of-life impact) contribute to one Problem Score and are not treated as separate dimensions. At least 15 paired items are required.

The submitted analysis listed eight construct-validity relationships because the two components of the Problem Score were correlated separately with the same two comparators. Once the validated Problem Score was restored, those four duplicate component-level entries became two score-level hypotheses. The resulting six nonredundant hypotheses preserve the original constructs, directions, and magnitude thresholds.

# Supplementary Table S1

Construct-validity hypotheses for the original Q-TFA score structure

| **Q-TFA score** | **Comparator** | **Expected association and rationale** | **Threshold** | **Observed ρ (95% CI)** | **Confirmed** |
| --- | --- | --- | --- | --- | --- |
| Prosthetic Use | PMQ 2.0 | Positive; both reflect prosthetic mobility/use | ρ≥0.50 | 0.69 (0.53 to 0.80) | Yes |
| Prosthetic Use | EQ VAS | Positive; greater use should relate to better self-rated health | ρ≥0.30 | 0.47 (0.24 to 0.64) | Yes |
| Problem | EQ-5D-5L profile severity | Positive; both are higher with more health problems | ρ≥0.50 | 0.66 (0.49 to 0.78) | Yes |
| Problem | SAT-PRO | Negative; more problems should relate to lower satisfaction | ρ≤-0.50 | -0.73 (-0.83 to -0.58) | Yes |
| Global | EQ VAS | Positive; both reflect favorable overall status | ρ≥0.30 | 0.51 (0.29 to 0.67) | Yes |
| Global | SAT-PRO | Positive; favorable overall status should relate to satisfaction | ρ≥0.30 | 0.40 (0.17 to 0.60) | Yes |

All 6/6 hypotheses were confirmed (100%); the prespecified supportive threshold was ≥75%.
